# Germline targeted next-generation sequencing in patients with adrenal incidentalomas

**DOI:** 10.3389/fendo.2025.1685220

**Published:** 2025-10-02

**Authors:** Erika Messina, Alessia Inglesi, Soraya Puglisi, Anja Barač Nekić, Valentina Morelli, Ylenia Alessi, Karin Zibar Tomsic, Serena Palmieri, Pasquale Tomaiuolo, Enrico Grosso, Francesco Ferraù, Iacopo Chiodini, Darko Kastelan, Anna Pia, Maria Scatolini, Giuseppe Reimondo, Massimo Terzolo

**Affiliations:** ^1^ Department of Clinical and Biological Sciences, Internal Medicine, San Luigi Gonzaga Hospital, University of Turin, Orbassano, Italy; ^2^ Molecular Oncology Laboratory, Fondazione Edo ed Elvo Tempia, Ponderano, Italy; ^3^ Department of Endocrinology, University Hospital Zagreb, Zagreb, Croatia; ^4^ Unit for Bone Metabolism Diseases and Diabetes and Lab of Endocrine and Metabolic Research, Istituto di Ricovero e Cura a Carattere Scientifico (IRCCS), Istituto Auxologico Italiano, Milan, Italy; ^5^ Department of Biomedical, Dental and Morphological and Functional Imaging Sciences, University of Messina, Messina, Italy; ^6^ Endocrinology Unit, Fondazione Istituto di Ricovero e Cura a Carattere Scientifico (IRCCS) Ca’ Granda-Ospedale Maggiore Policlinico, Milan, Italy; ^7^ Department of Human Pathology G. Barresi, Endocrine Unit, University Hospital G. Martino, University of Messina, Messina, Italy; ^8^ Department of Biotechnology and Translational Medicine, Unit of Endocrinology, Ospedale Niguarda Cà Granda, University of Milan, Milan, Italy; ^9^ Department of Endocrinology, Diabetes and Metabolism, Santa Croce and Carle Hospital, Cuneo, Italy

**Keywords:** genetics, variant, mutation, genotype-phenotype correlation, cortisol, adrenal adenoma, primary bilateral macronodular adrenal hyperplasia

## Abstract

**Objective:**

Adrenal incidentalomas are commonly detected in clinical practice. Despite growing interest in their molecular features, their germline genetic background remains largely unexplored. This study investigated the presence and potential pathogenic role of germline variants (GVs) in these patients using a targeted next-generation sequencing (NGS) approach, and explored possible genotype-phenotype correlations.

**Design:**

This multicenter retrospective study included 191 patients with incidentally discovered adrenal masses from four European reference centers. Patients with adrenocortical carcinoma, pheochromocytoma and primary aldosteronism were excluded.

**Methods:**

Germline DNA was extracted from peripheral blood and analyzed using a custom next-generation sequencing (NGS) panel targeting 21 genes potentially involved in adrenal tumorigenesis. Bioinformatic analysis was followed by variant classification using the ClinVar and VarSome databases, in accordance with ACMG guidelines.

**Results:**

GVs were identified in 12 of 191 patients (6.3%), affecting 7 different genes: ZNRF3, ARMC5, APC, CACNA1H, SCNN1B, PDE11A, and KCNJ5. Most of the detected variants were classified as variants of uncertain significance (VUS). Genotype-phenotype analysis revealed that some patients with GVs had bilateral adrenal lesions and/or mild autonomous cortisol secretion (MACS). No variants were classified as clearly pathogenic.

**Conclusion:**

This study provides new insights into the germline genetic landscape of adrenal incidentalomas. Although GVs were identified in a minority of patients, their clinical significance remains unclear due to the predominance of VUS. These findings do not currently support widespread germline testing in routine clinical management of adrenal incidentalomas. Nevertheless, the detection of potentially pathogenic variants may inform future studies exploring their possible role in adrenal tumorigenesis.

## Introduction

Adrenal tumors are a heterogeneous group of neoplasms in terms of frequency, clinical presentation, and outcomes. The group includes a wide spectrum of pathological entities, from the rare and aggressive adrenocortical carcinoma (ACC) to the frequent and benign adrenal adenoma (1). Currently, the majority of adrenal adenomas are serendipitously detected by imaging tests performed for unrelated reasons in up to 5-7% of people, and therefore they are called “adrenal incidentalomas” (2, 3).

Adrenal incidentalomas are most commonly non-functioning; however, overt cortisol or aldosterone hypersecretion occurs in approximately 15% of cases, while mild autonomous cortisol secretion (MACS) is observed in up to 30–50% of cases (4, 5). Malignant tumors are infrequent among adrenal incidentalomas but should not be underestimated since they accounted for 8.6% of all cases according to population-based data (6).

The molecular mechanisms underlying the occurrence of adrenocortical tumors are complex and not yet fully elucidated. Nevertheless, several key oncogenic pathways have been identified for adrenocortical carcinoma, such as *IGF*, *TP53* and the Wnt/β-catenin pathways (7–11). In benign adrenocortical adenomas, the activation of the cAMP/PKA signaling cascade has been frequently reported: somatic mutations in the catalytic alpha subunit of the protein kinase A (*PRKACA*) and the stimulatory G-protein alpha subunit (*GNAS*) are the most frequently observed alterations (12–14). These are driving factors in up to 35–60% of cortisol producing adenoma associated with overt Cushing syndrome, and are associated with a clinical phenotype characterized by more severe hormonal hypersecretion (15, 16). Conversely, these genetic variants are less frequently found in non-functioning adrenocortical adenoma or adenomas associated with MACS (12, 17–19). Indeed, the overall prevalence of *PRKACA* mutations in adenomas associated with MACS is less than 4.5%, while the most common genetic alteration is a somatic mutation of *CTNNB1* (encoding β-catenin) with a prevalence similar to that observed in non-functioning adenomas (17).

An increasing number of molecular events with pathogenetic role have been identified in aldosterone-secreting adrenocortical adenomas: almost all (90%) are due to somatic variants in genes encoding ion channels or transporters. Somatic variants of *KCNJ5* (potassium inwardly rectifying channel subfamily J member 5) are present in approximately 40% of aldosterone-producing adenomas (20). Other genes (*CACNA1D*, *ATP1A1*, *ATP2B3*, *CTNNB1*) are less frequently involved (21–23), and familial forms of primary aldosteronism due to germline mutations are uncommon.

The availability of next-generation sequencing (NGS) has boosted the study of genetic mechanisms and has facilitated the identification of both germline and somatic variants in human diseases, including adrenal disorders (21, 24). Although the genomic classification of benign adrenal lesions has been increasingly explored (21, 25–27), most studies have focused on somatic mutations in tumoral tissue (28), leaving the genetic background of adrenal incidentalomas still largely uncharted. This study aimed to identify germline mutations-associated with adrenal incidentalomas using a targeted sequencing approach and to explore potential genotype-phenotype correlations.

## Materials and methods

### Patient cohort

This multicenter study was conducted at four reference institutes which are part of the European Network for the Study of Adrenal Tumors (ENS@T): Orbassano (Turin), Milan, Messina (Italy) and Zagreb (Croatia). The study was conducted in accordance with the Declaration of Helsinki. Written informed consent was obtained from all participants, and the study was approved by the Local Ethics Committees. Patients with incidentally detected adrenal masses referred to the centers between 1999 and 2024 were retrospectively included in the study.

The inclusion criteria were as follows: age ≥18 years, incidentally detected tumor, presumed benign cortical adenoma, availability of a peripheral whole blood sample, and complete follow-up information. Diagnosis of adrenal adenoma was based on computed tomography (CT) features according to the guidelines of the time and later revised considering the current clinical practice guidelines (5, 29). In all patients, CT was repeated after 6 months to document unchanged CT features. Patients with radiological features suggesting ACC or with known extra-adrenal malignancies were excluded from the study. In agreement with the definition of adrenal incidentaloma, patients with severe or resistant hypertension, or hypokalemia or overt clinical signs of hypercortisolism were excluded. Catecholamine excess was excluded in all patients by measuring urinary fractionated metanephrines, while primary aldosteronism (PA) was excluded using the aldosterone-to-renin ratio (ARR), calculated from the pair plasma aldosterone concentration and plasma renin activity. Patients were considered negative for PA when their ARR values were below 30, in accordance with the guideline-recommended cutoff available at the time of study design (30).

Clinical and hormonal data were collected at diagnosis and at last follow-up. Clinical signs and symptoms of adrenal hormone overproduction were ruled out based on medical history and physical examination. The following data were obtained for further evaluation: age at the time of adrenal incidentaloma diagnosis, sex, family history of adrenal tumors, presence of arterial hypertension and glucose metabolism impairment, of any level. Hypertension and diabetes were either reported by the patient in the past medical history or diagnosed during the follow-up according to the guidelines available at the time (31, 32).

The measurement of urinary free cortisol (UFC), adrenocorticotropic hormone (ACTH), serum cortisol after overnight 1 mg dexamethasone suppression test (DST) were performed at baseline and at last follow-up. An incomplete cortisol suppression was defined by a post-DST cortisol level >1.8 μg/dL (50 nmol/L). Mild autonomous cortisol secretion (MACS) was considered for patients with an incomplete cortisol suppression following the DST without signs and symptoms of overt Cushing syndrome (5).

Next-Generation Sequencing (NGS) and bioinformatics analyses were conducted at the Molecular Oncology Laboratory, Edo and Elvo Tempia Foundation (Biella, Italy).

### NGS custom panel design

Targeted NGS was conducted using a custom panel (Illumina, San Diego, CA) targeting 21 genes potentially associated with benign adrenal tumors (*APC, AXIN1, ZNRF3, ARMC5, PRKAR1A, GNAS, CTNNB1, PRKACA, PRKACB, CACNA1H, SCNN1B, ATP1A1, KCNJ5, ATP2B3, CLCN2, DOT1L, HDAC9, NR3C1, PDE8B, PDE11A*, and *CYP11B2*) (**Supplementary Table S1**). The study protocol was conceived in 2019, when the targeted NGS panel was defined to include 21 genes selected based on the evidence available at the time and in accordance with the scope of the study that was approved by the local Ethics Committee. Given the subsequent delays in patient enrollment and laboratory processing (partly attributable to restrictions and work disruptions related to the COVID-19 pandemic) the initial gene panel was retained without modification to ensure consistency with the original design and Ethical clearance. Briefly, we included oncosuppressor genes involved in the regulation of the Wnt/B-catenin pathway in adrenal tumors and primary bilateral macronodular adrenal hyperplasia (PBMAH), as well as the cAMP-protein kinase A pathway (*APC*, *AXIN1*, *ZNRF3*, *ARMC5*, and *PRKAR1A*) (9, 33, 34). Additionally, genes associated with cancer-predisposition (*GNAS, CTNNB1, PRKACA, PRKACB*) (9, 35, 36) were added. Genes encoding proteins of membrane transport that are involved in the pathogenesis of aldosterone-producing adenomas (*CACNA1H, KCNJ5, SCNN1B, ATP1A1, ATP2B3, CLCN2*) (21–23) were also selected. Further, we included additional 6 genes encoding membrane receptors (*NR3C1*) (37, 38), enzymes (*PDE8B, PDE11A, CYP11B2*) (9, 21, 39) and proteins involved in the histone modification (*DOT1L, HDAC9*) (40). The custom panel was designed using the web-platform DesignStudio v.8.2.0.78 (https://www.illumina.com/products/by-type/informatics-products/designstudio.html) (Details in **Supplementary Table S2**). The selection of these genes was based on literature evidence highlighting their possible role in the pathogenesis of adrenal tumors (9, 25, 33, 34).

### DNA extraction from blood samples

Germline DNA was isolated from peripheral blood leukocytes in EDTA using QIAamp^®^ DNA Blood Mini-Kit (Qiagen, Hilden, Germany) according to the manufacturer’s instructions. DNA quantity of each sample was quantified by DeNovix DS-11 spectrophotometer (DeNovix Inc., Wilmington, DE, USA).

### NGS library preparation and sequencing for genomic germline DNA

DNA library preparation was conducted using the AmpliSeq Library Plus (Illumina, San Diego, CA, USA) following manufacturer’s instructions. Briefly, a total amount of 30 ng of genomic DNA per sample was used to generate amplicon libraries in a multiplex PCR amplification with two primer pools. The amplicons were partially digested and phosphorylated with FuPa reagent (Thermo Fisher Scientific), ligated to AmpliSeq CD indexes and purified with Agencourt^®^ AMPure^®^ XP beads (Beckman Coulter, Brea, CA, USA). The libraries were then amplified, purified with beads, and quantified on a Qubit^®^ 3.0 Fluorometer (Invitrogen, Waltham, MA, USA) using the dsDNA High Sensitivity kit. The pooled libraries were loaded for sequencing by synthesis on the Illumina MiSeq™ platform (Illumina, San Diego, CA, USA).

### NGS analysis and bioinformatics interpretation

A semi-automated bioinformatics pipeline was used. Bioinformatics analysis was performed with the DNA amplicon Analysis Module v2.1.0 workflow in Local Run Manager (Illumina, San Diego, CA, USA) through the following steps of analysis: demultiplexing and FASTQ generation, alignment to a reference genome and variant calling. The ANNOVAR tool (www.openbioinformatics.org/annovar/) was used to annotate all variants. Variant prioritization was obtained with a cascade of filtering. In particular, single-nucleotide polymorphisms (SNPs) ≥ 1% were excluded using data from the 1000Genome Project Data MAF (www.internationalgenome.org/) and subsequently with greater stringency based on the Non-Finnish European (NFE) category of the Genome Aggregation Database (gnomAD, https://gnomad.broadinstitute.org/). Furthermore, we removed synonymous variants that do not result in changes at the amino acid level. The identified variants were verified by the IGV (Integrative Genomics Viewer 92 v.2.5) (UC San Diego, Broad Institute).

Polymorphisms located in intronic regions were excluded from analysis. The pathogenic role of germline variants (GV) was assessed using the ClinVar database (https://www.ncbi.nlm.nih.gov/clinvar/) and the VarSome Premium tool (https://sso.varsome.com/) based on the American College of Medical Genetics and Genomics (ACMG) guidelines (41–43). Pathogenic (P), likely pathogenic (LP), variants of uncertain significance (VUS) or increased risk alleles variants were studied. Selected variants were validated through a Sanger sequencing (See **Supplementary Table S3**).

## Statistical analysis

Descriptive statistics were used to analyze the clinical variables. Continuous data are expressed as median and interquartile range (IQR). Categorical variables are indicated as numbers and percentages. Statistical analyses were performed with Jamovi - version 2.6.25.0.

## Results

### Patient characteristics

We evaluated 191 subjects, 127 females and 64 males (median age at the blood sample 58.2 years, IQR 33–82 years). None of them had a family history of adrenal diseases, thus all cases were apparently sporadic.

At diagnosis, 141 patients (74%) had a unilateral adrenal lesion, and 50 patients (26%) had bilateral masses. The average tumor size was 28.2 mm (IQR 20-33.5 mm). At diagnosis, 116 patients (61%) were hypertensive and 19 (10%) were diabetic. MACS, according to the criteria defined above, was detected in 46.6% (n=89) of patients, while non-functioning tumors were detected in 53.4% (n=102) of patients. The median follow-up was 8.5 years (IQR 3–15 years).

### Germline variants at NGS

Of the 191 patients, 12 (6.3%) had germline variants (GVs) in the panel of analyzed genes. A total of 12 unique GVs were identified in our patient cohort, including two identical variants in the *ARMC5* gene in two distinct carriers. One subject was carrier of two different variants (in *PDE11A* and *KCNJ5* genes respectively). These unique variants were identified in seven different genes: *ZNRF3* (n=2), *ARMC5* (n=1), *APC* (n=2), *CACNA1H* (n=4), *SCNN1B* (n=1), *PDE11A* (n=1), *KCNJ5* (n=1) (**Table 1**). Variants were interpreted following ClinVar classifications as follows: a) one (8%) variant of potential pathogenicity, classified as variant of uncertain significance/likely pathogenic (VUS/LP) in the *APC* gene; b) six (50%) VUS in *ARMC5* (n=1), *CACNA1H* (n=3), *PDE11A* (n=1), *SCNN1B* (n=1). Five out of 12 variants (42%) lack clinical evidence in ClinVar; therefore, the classification was implemented using the VarSome Premium tool, which applies the ACMG guidelines, resulting in: two VUS/LP variants in *ZNRF3* and *KCNJ5*; two VUS in *APC* and *CACNA1H*; and one variant in *ZNRF3* classified as benign (B). None GV was classified as pathogenic (P).

**Table 1:** Germline variants detected by NGS in a cohort of 191 patients with adrenal incidentalomas.

Pt ID	Gene (Ref. Seq)	Exon	Chr. position (hg19)^1^	HGVS.c	HGVS.p	Variant type	ClinVar (accessed on May2025)	VarSome	Gene function^2^
#1	*ZNRF3* (NM_001206998.1)	8	chr22:29446626	c.2460del	p.(Ala822ProfsTer4)	Frameshift deletion	N/A	B	Frizzled binding activity and ubiquitin-protein transferase activity
#2		8	chr22:29445997	c.1828_1829insGCT	p.(Ala610delinsGlySer)	Nonframeshift insertion	N/A	VUS/LP	
#3-4	*ARMC5* (NM_001105247.2)	6	chr16:31477594	c.2192C>G	p.(Pro731Arg) (p.P731R)	SNV	Conflicting interpretations of pathogenicity (3 VUS, 2 LB)	B	Protein binding. Modulation of steroidogenic enzymes expression
#5	*APC* (NM_000038.5)	16	chr5:112175211	c.3920T>A	p.(Ile1307Lys) (p.I1307K)	SNV	Conflicting interpretations of pathogenicity (5P, 9 LP, 5 RISK ALLELE, 11 VUS)	B	Protein binding Transcriptional activity, apoptosis regulation.
#6		16	chr5:112175211	c.6422G>C	p.(Gly2141Ala) (p.G2141A)	SNV	N/A	VUS	
#7	*CACNA1H* (NM_021098.2)	2	chr16:1203949	c.212C>G	p.(Pro71Arg) (p.P71R)	SNV	VUS	VUS	Voltage-gated monoatomic ion channel activity
#8		19	chr16:1260465	c.3941A>G	p.(Glu1314Gly) (p.E1314G)	SNV	N/A	VUS	
#9		10	chr16:1254253	c.2246C>T	p.(Thr749Met) (p.T749M)	SNV	VUS	LB	
#10		28	chr16:1265066	c.5024G>A	p.(Arg1675Gln) (p.R1675Q)	SNV	VUS	B	
#11	*SCNN1B* (NM_000336.3)	5	chr16:23379257	c.857C>T	p.(Ser286Leu) (p.S286L)	SNV	VUS	VUS	Ligand-gated Sodium channel activity
#12	*PDE11A* (NM_016953.3)	12	chr2:178592456	c.1973A>G	p.(Tyr658Cys) (p.Y658C)	SNV	VUS	LB	Signal transduction/Cyclic-nucleotide phosphodiesterase activity
#12	*KCNJ5* (NM_000890.3)	3	chr11:128786343	c.977A>G	p.(Glu326Gly) (p.E326G)	SNV	N/A	VUS/LP	Inward rectifier Potassium channel activity

^1^Assembly GRCh37/hg19; ^2^Gene function information was retrieved from GeneCards (www.genecards.org). Legend of abbreviations: chr, chromosome; HGVS, Human Genome Variation Society Nomenclature; SNV, single nucleotide variant; B, benign; LB, likely benign; VUS, variant of uncertain significance; LP, likely pathogenic; P, pathogenic; N/A, not available in database.

The complete list of germinal mutations including all the information about the type and localization of genetic alterations and the degree of pathogenicity classification is summarized in **Table 1**.

### Genotype-phenotype analysis

Clinical features of patients are detailed in **Table 2**.

**Table 2:** Clinical characteristics of patients with GVs.

Pt ID	Gene	Gender	Age at diagnosis (years)	Size of adrenal mass (mm)	Laterality	Diagnosis	Clinical phenotype
#1	ZNRF3	F	49	20	Unilateral	MACS	Hypertension
#2		F	38	18	Unilateral	NFAT	–
#3	ARMC5	M	56	22	Bilateral	MACS	Hypertension, IGT
#4		M	48	30	Bilateral	NFAT	Hypertension. IGT
#5	APC	F	62	16	Unilateral at diagnosis, bilateral at LFU	NFAT	–
#6		M	61	59	Bilateral	MACS	Hypertension
#7	CACNA1H	F	58	48	Bilateral	MACS	Hypertension
#8		F	40	40	Bilateral	NFAT	–
#9		F	70	50	Unilateral	MACS	Hypertension
#10		F	64	30	Bilateral	MACS	Hypertension, T2DM
#11	SCNN1B	F	49	25	Unilateral	MACS	Hypertension
#12	PDE11A KCNJ5	M	66	14	Unilateral	NFAT	Hypertension, T2DM

MACS, mild autonomous cortisol secretion; NFAT, nonfunctioning adrenal tumor; IGT, impaired glucose tolerance; T2DM, Type 2 Diabetes Mellitus; LFU, last follow-up.

Both female patients with GVs in the *ZNRF3* gene (c.2460delC, p.Ala822ProfsTer4 and c.1828_1829insGCT, p.Ala610delinsGlySer) had unilateral small adrenal lesions that did not change their CT characteristics during a mean follow-up of 7 years; however, the patients became hypertensive despite the absence of mineralocorticoid and glucocorticoid excess.

A unique GV c.2192C>G, p.(Pro731Arg) in exon 6 of the *ARMC5* gene was found in two male subjects. Both had bilateral lesions at diagnosis (the largest size of 22 mm and 30 mm, respectively) that did not grow significantly over time. One of them had hypertension and MACS at diagnosis, while the other became hypertensive during follow-up despite the absence of cortisol autonomy.

A 62-year-old woman, carrier of a well-known GV in exon 16 of the *APC* gene c.3920T>A, (p.Ile1307Lys) (I1307K), presented with a non-secreting adrenal lesion of 16 mm. During follow-up she developed an adenoma with similar characteristics in the contralateral gland. Instead, a novel GV c.6422G>C, p.(Gly2141Ala) in the *APC* gene was found in a 61-year-old man with bilateral adrenal lesions (the largest of 59 mm) and MACS.

Three of four GVs in the *CACNA1H* gene were classified as VUS based on the ClinVar database (ID: #7, c.C212G; #9, c.2246C>T; #10, c.5024G>A) and were not associated with aldosterone hypersecretion. However, the three women who carried these VUS (#7, #9 and #10) were all hypertensive and diagnosed with MACS associated with bilateral adrenal lesions. The other patient (ID: 8#, c.3941A>G) displayed a unilateral adrenal mass with a size of 40 mm, which remained stable over time, with no occurrence of hypertension and no evidence of excess hormone secretion.

A 49-years old woman with a GV c.857C>T, p.(Ser286Leu) in the *SCNN1B* gene presented a single adrenal lesion with MACS and developed hypertension overtime.

GVs in both *PDE11A* and *KCNJ5* genes were identified in a hypertensive, diabetic male patient presenting with a 14 mm non-secreting adrenal mass, which demonstrated no radiological progression over a 7-year follow-up period.

## Discussion

Adrenal tumors occur sporadically, and only in rare instances in the context of familial genetic syndromes. This clinical observation supports the view that hereditable genetic factors contribute to their etiopathogenesis in a minority of cases (44). To date, genetic research has primarily focused on patients with primary aldosteronism (45, 46), or Cushing syndrome (47, 48) or ACC (49, 50). Clinically non-functioning adrenal incidentalomas received less attention. This multicenter study represents, to our knowledge, one of the few studies aimed to investigate the germline variants associated with adrenal incidentalomas using a targeted sequencing approach and to explore potential genotype-phenotype correlations.

Targeted NGS focused on 21 genes whose selection was based on literature data supporting their potential role in adrenal tumor formation. Our findings show that GVs in genes implicated in adrenal tumorigenesis are present in approximately 6.3% of patients with adrenal incidentaloma. Currently, the clinical significance of these variants remains uncertain, as the majority are classified as VUS according to the ClinVar database. Nonetheless, these VUS could be reclassified based on new evidence thanks to functional assays or emerging clinical data. Therefore, a potential pathogenic role for these variants cannot be ruled out at this time. In summary, we identified GVs in only 7 of the 21 genes analyzed in the panel, *ZNRF3, ARMC5, APC, CACNA1H, SCNN1B, PDE11A, KCNJ5.*

Somatic mutations of ZNRF3 are present in approximately 20% of patients with ACC (51), a finding that suggests an important role in tumor development through aberrant activation of the Wnt pathway (7, 8). In contrast, such mutations are rarely observed in adrenal adenomas (17, 25) suggesting a limited pathogenic role in benign lesions. In our series, we observed two GVs of the *ZNRF3* gene in patients with adrenal adenomas lacking any suspicious features and remaining radiologically stable over time. Both identified tumor suppressor variants -a novel in-frame variant p.(Ala610delinsGlySer) and a frameshift variant p.(Ala822ProfsTer4)- have not been previously reported in the ClinVar database. While somatic mutations in *ZNRF3* are commonly observed across various cancers, the implications of germline variations in this gene remain less well understood. Notably, the p.(Ala610delinsGlySer) variant is classified in the Varsome Premium tool as benign, based on its allele frequency in the gnomAD exome dataset (0.000164), and its presence primarily in healthy adult individuals. This alteration results in a change in the protein coding sequence length and does not occur within a known repeat region, suggesting that it may not arise from replication slippage or repeat-associated mutational mechanisms (52). Furthermore, we identified a novel null variant in the *ZNRF3* gene (c.2460del), leading to a frameshift and premature stop codon. Although this variant has not yet been reported in ClinVar nor established as a germline contributor to adrenal incidentalomas, Varsome Premium classifies it as a variant of VUS/LP, due to a frameshift mutation presumed to cause nonsense-mediated decay. Importantly, *ZNRF3* inactivation has been demonstrated to cause hyperactivation of Wnt/β-catenin signaling, a pathway critical for regulating cell growth, differentiation, and tumor development (51, 53). Nevertheless, to date, no studies have explored the clinical implications of these variants in patients with adrenal incidentalomas.

Inactivating mutations in *ARMC5* are the most common genetic alterations in PBMAH, accounting for approximately 80% of familial and 20–25% of sporadic cases (10, 54, 55). Pathogenic GVs in *ARMC5* have already been detected in 18.8% of patients with bilateral adrenal incidentalomas and MACS (56). The heterozygous p.(Pro731Arg) variant in *ARMC5*, currently classified as conflicting interpretation, has been reported in earlier studies, indicating that it represents a relatively frequent observation among reported cases (10, 50, 55, 56). We found this variant in two patients with bilateral adrenal lesions (and MACS in one of them). The relative frequency of non-pathogenic *ARMC5* variants suggests that multiple molecular mechanisms are needed in PBMAH pathogenesis (57). This finding leads also to the speculation that a continuum may exist between bilateral adenomas and PBMAH and that what we currently interpret as bilateral adenomas might represent early stages of PBMAH. PBMAH frequently results from heterozygous germline inactivating mutations, often accompanied by a second somatic mutation, consistent with a ‘two-hit’ model of tumorigenesis (33). However, recent studies have identified somatic *ARMC5* mutations occurring independently of germline alterations (56). A potential limitation of our study is the lack of genetic analysis on adrenal tissue, which precludes the assessment of somatic mutations role. Nonetheless, the recurrence of the ARMC5 variants in patients with bilateral adrenal tumors across different cohorts, including ours, supports the existence of a definitive genotype–phenotype correlation. In line with this, ARMC5 testing may be most informative in patients with bilateral adrenal lesions, especially when associated with MACS, as already proposed by Mariani et al. (2020) (58).

We identified two different GVs of the *APC* gene. Other studies investigated the natural history of adrenal incidentalomas in familial adenomatous polyposis (FAP) and found that these lesions typically exhibit a non-aggressive behavior (59). Of particular interest, the *APC* p.(Ile1307Lys) variant is a missense polymorphism with an allele frequency of 0.002008 (gnomAD exome) in the general population, indicating that it is relatively common and should therefore be considered of limited clinical significance. In particular, this variant is a low-to-moderate penetrance alteration associated with a moderately increased risk of colorectal cancer exclusively in individuals of Ashkenazi Jewish origin (60–62). However, the InSight consortium has highlighted challenges in applying standard variant interpretation frameworks to this peculiar alteration, leading to inconsistencies in its classification (63). Currently, there is insufficient high-quality evidence to establish the p.(Ile1307Lys) variant as a significant risk factor for colorectal cancer in non-Ashkenazi Jewish populations or for extracolonic malignancies (64).

We identified another *APC* variant p.(Gly2164Ala) in a male patient presenting with bilateral adrenal lesions and MACS, but without evidence of colonic polyposis. This specific variant has not previously been documented in individuals with APC-related syndromes, nor is it reported in population databases such as the gnomAD, indicating that it is a rare or possibly novel alteration. Currently, there is insufficient data to clarify the pathogenicity or clinical significance of this variant. Mutations in the *APC* gene, particularly those affecting exon 16, are well-established contributors to colorectal carcinogenesis (65). Especially, exon 16 is the largest exon, encoding approximately 77% of the *APC* protein and encompassing multiple critical functional domains (63). It has been observed that the majority (~94%) of germline APC mutations result in truncated proteins, for instance caused by nonsense or frameshift mutations (66).

Literature data showed that both somatic and germline heterozygous mutations in *CACNA1H*, which encodes the alpha-1 subunit family of T-type calcium channel (CaV3.2), result in a gain-of-function effect, leading to enhanced aldosterone synthesis (22). In our cohort, primary aldosteronism was excluded at diagnosis; nonetheless, three of the four carriers of these variants of uncertain clinical significance in *CACNA1H* presented with hypertension and MACS. This finding is in line with the concept that autonomous aldosterone secretion extends beyond the traditional boundaries of primary aldosteronism (67) and reinforce the notion of a continuum, in which mild or subclinical forms may not be reliably captured by standard screening approaches. In the fourth carrier, *CACNA1H* SNV p.(Glu1314Gly) was identified, which has not yet been classified in the ClinVar database. According to Varsome, this variant is currently categorized as of uncertain significance while multiple *in silico* prediction tools suggest a likely deleterious effect (SIFT: 0.001; PolyPhen-2: 1.0; FATHMM: -4.55). This amino acid substitution occurs within the homologous domain III transmembrane α-helix, a region known to play a key role in regulating calcium ion flux through the CaV3.2 channel, thereby influencing cellular processes (68). The replacement of a negatively charged glutamic acid with a neutral glycine could disrupt the structural stability of the channel protein, potentially impairing its folding and function. Although these bioinformatic predictions raise concerns about its pathogenic potential, further functional validation is required to determine the true clinical significance of this variant in the context of adrenal incidentalomas.

Mutations in *SCNN1B*, belonging to the sodium channel genes, may cause a rare dominant form of monogenic hypertension. We found the p.(Ser286Leu) variant in a patient with MACS. However, this variant has been previously reported as benign (69). Functional studies on this variant, which is located in exon 5, suggest that it does not significantly alter the cellular sodium transport activity (70).

Defects in the PDE11A gene, encoding phosphodiesterase type 11, represent the main genetic cause of isolated primary pigmented nodular dysplasia of the adrenal cortex (iPPNAD), and are also detectable in PBMAH and adrenocortical adenomas (71). The p.(Tyr658Cys) variant, previously identified in a patient with ACTH-dependent macronodular adrenal hyperplasia (72), lies outside canonical functional domain. Although not located within the catalytic or regulatory regions, *in silico* algorithms SIFT and Polyphen-2 predict this missense substitution could be deleterious. Functional studies further support its significant impact on enzyme activity: transfection experiments in different cell lines demonstrated markedly reduced PDE activity associated with the Y658C variant, with the most pronounced inhibitory effect among the tested mutations (73). This reduction in enzyme activity likely inhibits intracellular cAMP and cGMP regulation, key pathways in adrenal cortex homeostasis. The study of Faucz et al. (2011) also reported that the observed differences in variant behavior between cell types suggest tissue-specific effects, which may underlie the clinical variability observed among patients carrying *PDE11A* mutations (73). Further in-depth functional and clinical studies are needed to definitively establish its pathogenic role.

GVs of *KCNJ5* are implicated in rare syndromic forms of primary aldosteronism, unlike its somatic mutations which are often causative of aldosterone-producing adenomas (74). The p.(Glu326Gly) variant falls within a functional domain present in the third coding exon. The effect of amino acid substitution within the inward-rectifying potassium (K) channels domain is well studied, and the PA-associated mutations in *KCNJ5* typically cause a marked reduction in K+ selectivity (75, 76). Hence the channels carry a significant inward Na+ current, which is thought to depolarize the zona glomerulosa cells triggering increased aldosterone synthesis and release. *In silico* predictors suggest that it could be deleterious; however, there are no studies reporting this specific mutation. Notably, our subject carrying both PDE11A and KCNJ5 variants presented with hypertension and a non-functioning adrenal tumor.

In conclusion, this study provides the first germline-level analysis in a large cohort of patients with adrenal incidentalomas to identify genetic variants in potential predisposition genes. In our cohort, no GV with a current clearly established pathogenic role was identified. Most of the variants found in our cohort were variants of uncertain significance (VUS), a classification that is likely due to the limited data available in the current literature. We disclose the limit that the use of a targeted panel in this study could be associated with a limited diagnostic yield, highlighting the need for broader genomic strategies in future cohorts. Larger, adrenal-disease–inclusive panels or whole-exome sequencing could help uncover rare or unexpected predisposition genes, provide a more comprehensive framework to assess genotype–phenotype correlations, and facilitate a deeper understanding of the molecular basis of adrenal incidentalomas. To clarify the potential pathogenicity of the variants of currently unknown significance, further functional studies correlating genetic findings with clinical phenotypes will be necessary. Given the current status of knowledge, the present findings suggest that germline testing is not useful in the management of patients with adrenal incidentalomas, and widespread germline testing may therefore not be warranted.

## Data Availability

The original contributions presented in the study are included in the article/**Supplementary Material**, further inquiries can be directed to the corresponding author/s.
