# Supplementary material for: Germline targeted next-generation sequencing in patients with adrenal incidentalomas
Source: Front Endocrinol (Lausanne). 2025 Oct 2;16:1685220. doi: 10.3389/fendo.2025.1685220 (PMC12527831; doi:10.3389/fendo.2025.1685220)
Supplement: Supplementary file 1 [file DataSheet1.pdf]

Table S1. *List of the genes included in the NGS custom panel.*

Gene	Description	CytoBand	OMIM	Summary
APC	APC regulator of WNT signaling pathway	5q22.2	175100 Other diseases (114500, 135290, 619182,613659,114550)	This gene encodes a tumor suppressor protein that acts as an antagonist of the Wnt signaling pathway. It is also involved in other processes including cell migration and adhesion, transcriptional activation, and apoptosis. Defects in this gene cause familial adenomatous polyposis (FAP), an autosomal dominant pre-malignant disease that usually progresses to malignancy. Mutations in the APC gene have been found to occur in most colorectal cancers, where disease-associated mutations tend to be clustered in a small region designated the mutation cluster region (MCR) and result in a truncated protein product.
ARMC5	Armadillo repeat containing 5	16p11.2	615954	This gene encodes a member of the ARM (armadillo/beta-catenin-like repeat) superfamily. The ARM repeat is a tandemly repeated sequence motif with approximately 40 amino acid long. This repeat is implicated in mediating protein-protein interactions. The encoded protein contains seven ARM repeats. Mutations in this gene are associated with primary bilateral macronodular adrenal hyperplasia, which is also known as ACTH-independent macronodular adrenal hyperplasia 2.
ATP1A1	ATPase Na+/K+ transporting subunit alpha 1	1p13.1	618036 Other diseases (618314)	The protein encoded by this gene belongs to the family of P-type cation transport ATPases, and to the subfamily of Na+/K+ -ATPases. Na+/K+ -ATPase is an integral membrane protein responsible for establishing and maintaining the electrochemical gradients of Na and K ions across the plasma membrane. These gradients are essential for osmoregulation, for sodium-coupled transport of a variety of organic and inorganic molecules, and for electrical excitability of nerve and muscle. This enzyme is composed of two subunits, a large catalytic subunit (alpha) and a smaller glycoprotein subunit (beta). The catalytic subunit of Na+/K+ -ATPase is encoded by multiple genes. This gene encodes an alpha 1 subunit.
ATP2B3	ATPase plasma membrane Ca2+ transporting 3	Xq28	302500	The protein encoded by this gene belongs to the family of P-type primary ion transport ATPases characterized by the formation of an aspartyl phosphate intermediate during the reaction cycle. These enzymes remove bivalent calcium ions from eukaryotic cells against very large concentration gradients and play a critical role in intracellular calcium homeostasis. The mammalian plasma membrane calcium ATPase isoforms are encoded by at least four separate genes and the diversity of these enzymes is further increased by alternative splicing of transcripts. The expression of different isoforms and splice variants is regulated in a developmental, tissue- and cell type-specific manner, suggesting that these pumps are functionally adapted to the physiological needs of particular cells and tissues. This gene encodes the plasma membrane calcium ATPase isoform 3

AXIN1	Axin 1	16p13.3	607864 Other diseases (114550)	This gene encodes a cytoplasmic protein which contains a regulation of G-protein signaling (RGS) domain and a dishevelled and axin (DIX) domain. The encoded protein interacts with adenomatosis polyposis coli, catenin beta-1, glycogen synthase kinase 3 beta, protein phosphate 2, and itself. This protein functions as a negative regulator of the wingless-type MMTV integration site family, member 1 (WNT) signaling pathway and can induce apoptosis. The crystal structure of a portion of this protein, alone and in a complex with other proteins, has been resolved. Mutations in this gene have been associated with hepatocellular carcinoma, hepatoblastomas, ovarian endometriod adenocarcinomas, and medullablastomas.
CACNA1H	Calcium voltage-gated channel subunit alpha1 H	16p13.3	617027 Other diseases (611942)	This gene encodes a T-type member of the alpha-1 subunit family, a protein in the voltage-dependent calcium channel complex. Calcium channels mediate the influx of calcium ions into the cell upon membrane polarization and consist of a complex of alpha-1, alpha-2/delta, beta, and gamma subunits in a 1:1:1:1 ratio. The alpha-1 subunit has 24 transmembrane segments and forms the pore through which ions pass into the cell. There are multiple isoforms of each of the proteins in the complex, either encoded by different genes or the result of alternative splicing of transcripts. Alternate transcriptional splice variants, encoding different isoforms, have been characterized for the gene described here. Studies suggest certain mutations in this gene lead to childhood absence epilepsy (CAE).
CLCN2	Chloride voltage-gated channel 2	3q27.1	605635 Other diseases (607628, 615651)	This gene encodes a voltage-gated chloride channel. The encoded protein is a transmembrane protein that maintains chloride ion homeostasis in various cells. Defects in this gene may be a cause of certain epilepsies. Four transcript variants encoding different isoforms have been found for this gene.
CTNNB1	Catenin beta 1	3p22.1	114500 Other diseases (617572, 114550, 155255, 615075, 167000, 132600)	The protein encoded by this gene is part of a complex of proteins that constitute adherens junctions (AJs). AJs are necessary for the creation and maintenance of epithelial cell layers by regulating cell growth and adhesion between cells. The encoded protein also anchors the actin cytoskeleton and may be responsible for transmitting the contact inhibition signal that causes cells to stop dividing once the epithelial sheet is complete. Finally, this protein binds to the product of the APC gene, which is mutated in adenomatous polyposis of the colon. Mutations in this gene are a cause of colorectal cancer (CRC), pilomatrixoma (PTR), medulloblastoma (MDB), and ovarian cancer.

CYP11B2	Cytochrome P450 family 11 subfamily B member 2	8q24.3	203400 Other diseases (610600)	This gene encodes a member of the cytochrome P450 superfamily of enzymes. The cytochrome P450 proteins are monooxygenases which catalyze many reactions involved in drug metabolism and synthesis of cholesterol, steroids and other lipids. This protein localizes to the mitochondrial inner membrane. The enzyme has steroid 18-hydroxylase activity to synthesize aldosterone and 18-oxocortisol as well as steroid 11 beta-hydroxylase activity. Mutations in this gene cause corticosterone methyl oxidase deficiency.
DOT1L	DOT1 like histone lysine methyltransferase	19p13.3	607375	The protein encoded by this gene is a histone methyltransferase that methylates lysine-79 of histone H3. It is inactive against free core histones, but shows significant histone methyltransferase activity against nucleosomes.
GNAS	GNAS complex locus	20q13.32	219080 Other diseases (174800, 166350, 617686, 103580, 603233, 612462, 612463)	This locus has a highly complex imprinted expression pattern. It gives rise to maternally, paternally, and biallelically expressed transcripts that are derived from four alternative promoters and 5' exons. Some transcripts contain a differentially methylated region (DMR) at their 5' exons, and this DMR is commonly found in imprinted genes and correlates with transcript expression. An antisense transcript is produced from an overlapping locus on the opposite strand. One of the transcripts produced from this locus, and the antisense transcript, are paternally expressed noncoding RNAs, and may regulate imprinting in this region. In addition, one of the transcripts contains a second overlapping ORF, which encodes a structurally unrelated protein - Alex. Alternative splicing of downstream exons is also observed, which results in different forms of the stimulatory G-protein alpha subunit, a key element of the classical signal transduction pathway linking receptor-ligand interactions with the activation of adenylyl cyclase and a variety of cellular reponses. Multiple transcript variants encoding different isoforms have been found for this gene. Mutations in this gene result in pseudohypoparathyroidism type 1a, pseudohypoparathyroidism type 1b, Albright hereditary osteodystrophy, pseudopseudohypoparathyroidism, McCune-Albright syndrome, progressive osseus heteroplasia, polyostotic fibrous dysplasia of bone, and some pituitary tumors.
HDAC9	Histone deacetylase 9	7p21.1	606543	Histones play a critical role in transcriptional regulation, cell cycle progression, and developmental events. Histone acetylation/deacetylation alters chromosome structure and affects transcription factor access to DNA. The protein encoded by this gene has sequence homology to members of the histone deacetylase family. This gene is orthologous to the Xenopus and mouse MITR genes. The MITR protein lacks the histone deacetylase catalytic domain. It represses MEF2 activity through recruitment of multicomponent corepressor complexes that include CtBP and HDACs. This encoded protein may play a role in hematopoiesis.

KCNJ5	Potassium inwardly rectifying channel subfamily J member 5	11q24.3	613677 Other diseases (613485)	This gene encodes an integral membrane protein which belongs to one of seven subfamilies of inward-rectifier potassium channel proteins called potassium channel subfamily J. The encoded protein is a subunit of the potassium channel which is homotetrameric. It is controlled by G-proteins and has a greater tendency to allow potassium to flow into a cell rather than out of a cell. Naturally occurring mutations in this gene are associated with aldosterone-producing adenomas
NR3C1	Nuclear receptor subfamily 3 group C member 1	5q31.3	615962	This gene encodes glucocorticoid receptor, which can function both as a transcription factor that binds to glucocorticoid response elements in the promoters of glucocorticoid responsive genes to activate their transcription, and as a regulator of other transcription factors. This receptor is typically found in the cytoplasm, but upon ligand binding, is transported into the nucleus. It is involved in inflammatory responses, cellular proliferation, and differentiation in target tissues. Mutations in this gene are associated with generalized glucocorticoid resistance. Alternative splicing of this gene results in transcript variants encoding either the same or different isoforms. Additional isoforms resulting from the use of alternate in-frame translation initiation sites have also been described, and shown to be functional, displaying diverse cytoplasm-to-nucleus trafficking patterns and distinct transcriptional activities
PDE11A	Phosphodiesterase 11A	2q31.2	610475	The 3',5'-cyclic nucleotides cAMP and cGMP function as second messengers in a wide variety of signal transduction pathways. 3',5'-cyclic nucleotide phosphodiesterases (PDEs) catalyze the hydrolysis of cAMP and cGMP to the corresponding 5'-monophosphates and provide a mechanism to downregulate cAMP and cGMP signaling. This gene encodes a member of the PDE protein superfamily. Mutations in this gene are a cause of Cushing disease and adrenocortical hyperplasia.
PDE8B	Phosphodiesterase 8B	5q13.3	614190 Other diseases (609161)	The protein encoded by this gene is a cyclic nucleotide phosphodiesterase (PDE) that catalyzes the hydrolysis of the second messenger cAMP. The encoded protein, which does not hydrolyze cGMP, is resistant to several PDE inhibitors. Defects in this gene are a cause of autosomal dominant striatal degeneration (ADSD).

PRKACA	Protein kinase cAMP-activated catalytic subunit alpha	19p13.12	601639 other disease (619142, 615830)	This gene encodes one of the catalytic subunits of protein kinase A, which exists as a tetrameric holoenzyme with two regulatory subunits and two catalytic subunits, in its inactive form. cAMP causes the dissociation of the inactive holoenzyme into a dimer of regulatory subunits bound to four cAMP and two free monomeric catalytic subunits. Four different regulatory subunits and three catalytic subunits have been identified in humans. cAMP-dependent phosphorylation of proteins by protein kinase A is important to many cellular processes, including differentiation, proliferation, and apoptosis. Constitutive activation of this gene caused either by somatic mutations, or genomic duplications of regions that include this gene, have been associated with hyperplasias and adenomas of the adrenal cortex and are linked to corticotropin-independent Cushing's syndrome. Alternative splicing results in multiple transcript variants encoding different isoforms. Tissue-specific isoforms that differ at the N-terminus have been described, and these isoforms may differ in the post-translational modifications that occur at the N-terminus of some isoforms.
PRKACB	Protein kinase cAMP-activated catalytic subunit beta	1p31.1	176892 other disease (619143)	The protein encoded by this gene is a member of the serine/threonine protein kinase family. The encoded protein is a catalytic subunit of cAMP (cyclic AMP)-dependent protein kinase, which mediates signalling though cAMP. cAMP signaling is important to a number of processes, including cell proliferaton and differentiation. Multiple alternatively spliced transcript variants encoding distinct isoforms have been observed.
PRKAR1A	Protein kinase cAMP-dependent type I regulatory subunit alpha	17q24.2	264350 Other diseases (211400,177200)	cAMP is a signaling molecule important for a variety of cellular functions. cAMP exerts its effects by activating the cAMP-dependent protein kinase, which transduces the signal through phosphorylation of different target proteins. The inactive kinase holoenzyme is a tetramer composed of two regulatory and two catalytic subunits. cAMP causes the dissociation of the inactive holoenzyme into a dimer of regulatory subunits bound to four cAMP and two free monomeric catalytic subunits. Four different regulatory subunits and three catalytic subunits have been identified in humans. This gene encodes one of the regulatory subunits. This protein was found to be a tissue-specific extinguisher that down-regulates the expression of seven liver genes in hepatoma x fibroblast hybrids. Mutations in this gene cause Carney complex (CNC). This gene can fuse to the RET protooncogene by gene rearrangement and form the thyroid tumor-specific chimeric oncogene known as PTC2. A nonconventional nuclear localization sequence (NLS) has been found for this protein which suggests a role in DNA replication via the protein serving as a nuclear transport protein for the second subunit of the Replication Factor C (RFC40)

SCNN1B	Sodium channel epithelial 1 subunit beta	16p12.2	612062	Nonvoltage-gated, amiloride-sensitive, sodium channels control fluid and electrolyte transport across epithelia in many organs. These channels are heteromeric complexes consisting of 3 subunits: alpha, beta, and gamma. This gene encodes the beta subunit, and mutations in this gene have been associated with pseudohypoaldosteronism type 1 (PHA1), and Liddle syndrome.
ZNRF3	Zinc and ring finger 3	22q12.1	188830	Enables frizzled binding activity and ubiquitin-protein transferase activity. Involved in negative regulation of Wnt signaling pathway; protein ubiquitination; and ubiquitin-dependent protein catabolic process. Located in plasma membrane.
