# Supplementary material for: Germline targeted next-generation sequencing in patients with adrenal incidentalomas
Source: Front Endocrinol (Lausanne). 2025 Oct 2;16:1685220. doi: 10.3389/fendo.2025.1685220 (PMC12527831; doi:10.3389/fendo.2025.1685220)
Supplement: Supplementary file 2 [file DataSheet2.pdf]

Table S2. Details of the NGS targeted panel

Target_ID	Gene_Symbol	Chromosome	Start	End	Num_Amplicons	Total_Bases	Covered_Bas	Missed_Base	Coverage
58466	AXIN1	chr16	338097	338273	2	177	177	0	1
58459	AXIN1	chr16	339415	339632	2	218	218	0	1
58464	AXIN1	chr16	341165	341322	2	158	158	0	1
58467	AXIN1	chr16	343463	343743	3	281	281	0	1
58463	AXIN1	chr16	347031	347251	2	221	221	0	1
58462	AXIN1	chr16	347697	348276	4	580	580	0	1
58460	AXIN1	chr16	354279	354466	1	188	188	0	1
58465	AXIN1	chr16	359948	360094	2	147	147	0	1
58461	AXIN1	chr16	364518	364708	2	191	191	0	1
58458	AXIN1	chr16	396123	397050	6	928	928	0	1
129259	CACNA1H	chr16	1203713	1204061	4	349	349	0	1
67440	CACNA1H	chr16	1244947	1245108	1	162	162	0	1
67433	CACNA1H	chr16	1245407	1245590	1	184	184	0	1
67430	CACNA1H	chr16	1245901	1246048	2	148	148	0	1
67436	CACNA1H	chr16	1248590	1248799	2	210	210	0	1
67427	CACNA1H	chr16	1250231	1250596	3	366	366	0	1
67413	CACNA1H	chr16	1250691	1250833	2	143	143	0	1
67445	CACNA1H	chr16	1251638	1252477	5	840	840	0	1
67422	CACNA1H	chr16	1253985	1254483	3	499	499	0	1
67444	CACNA1H	chr16	1255089	1255290	2	202	202	0	1
67420	CACNA1H	chr16	1256079	1256314	2	236	236	0	1
67439	CACNA1H	chr16	1256976	1257143	2	168	168	0	1
67435	CACNA1H	chr16	1257250	1257455	1	206	206	0	1
67441	CACNA1H	chr16	1257745	1257885	1	141	141	0	1
67443	CACNA1H	chr16	1257988	1258246	2	259	259	0	1
67434	CACNA1H	chr16	1259007	1259437	3	431	431	0	1
67428	CACNA1H	chr16	1260010	1260160	2	151	151	0	1
67425	CACNA1H	chr16	1260345	1260518	2	174	174	0	1

67442	CACNA1H	chr16	1260558	1260676	2	119	119	0	1
67419	CACNA1H	chr16	1260762	1260996	2	235	235	0	1
67424	CACNA1H	chr16	1261143	1261319	2	177	177	0	1
67414	CACNA1H	chr16	1261456	1261631	2	176	176	0	1
67429	CACNA1H	chr16	1261691	1261830	2	140	140	0	1
67423	CACNA1H	chr16	1261921	1262163	2	243	243	0	1
67426	CACNA1H	chr16	1262486	1262553	1	68	68	0	1
67421	CACNA1H	chr16	1263755	1263956	2	202	202	0	1
67416	CACNA1H	chr16	1264947	1265106	2	160	160	0	1
67417	CACNA1H	chr16	1265217	1265400	2	184	184	0	1
67415	CACNA1H	chr16	1265498	1265618	2	121	121	0	1
67431	CACNA1H	chr16	1266907	1267035	2	129	129	0	1
67412	CACNA1H	chr16	1267894	1268065	2	172	172	0	1
67438	CACNA1H	chr16	1268185	1268676	4	492	492	0	1
67437	CACNA1H	chr16	1268945	1269155	2	211	211	0	1
129258	CACNA1H	chr16	1269956	1271019	7	1064	1064	0	1
248232	DOT1L	chr19	2164159	2164289	2	131	131	0	1
248214	DOT1L	chr19	2180687	2180780	1	94	94	0	1
248235	DOT1L	chr19	2185829	2185953	1	125	119	6	0,952
248218	DOT1L	chr19	2189706	2189819	1	114	114	0	1
248231	DOT1L	chr19	2190986	2191264	3	279	279	0	1
248219	DOT1L	chr19	2193663	2193807	2	145	145	0	1
248233	DOT1L	chr19	2194489	2194601	2	113	113	0	1
248220	DOT1L	chr19	2199858	2199963	1	106	106	0	1
248224	DOT1L	chr19	2202674	2202803	1	130	130	0	1
248237	DOT1L	chr19	2206703	2206821	1	119	119	0	1
248215	DOT1L	chr19	2207548	2207704	2	157	157	0	1
248229	DOT1L	chr19	2208909	2209000	1	92	92	0	1
248217	DOT1L	chr19	2210374	2210534	2	161	161	0	1
248228	DOT1L	chr19	2210595	2210879	3	285	285	0	1

248234	DOT1L	chr19	2211073	2211236	1	164	164	0	1
248227	DOT1L	chr19	2211725	2211866	1	142	142	0	1
248239	DOT1L	chr19	2213513	2213664	2	152	152	0	1
248216	DOT1L	chr19	2213823	2214010	2	188	188	0	1
248221	DOT1L	chr19	2214445	2214620	2	176	176	0	1
248236	DOT1L	chr19	2216255	2216789	3	535	535	0	1
248222	DOT1L	chr19	2216929	2217114	2	186	186	0	1
248213	DOT1L	chr19	2217746	2217942	2	197	197	0	1
248226	DOT1L	chr19	2220082	2220246	2	165	165	0	1
248230	DOT1L	chr19	2221950	2222583	4	634	634	0	1
248238	DOT1L	chr19	2223255	2223510	2	256	256	0	1
248223	DOT1L	chr19	2225362	2225476	1	115	115	0	1
248225	DOT1L	chr19	2226157	2227151	6	995	995	0	1
248212	DOT1L	chr19	2229759	2229816	2	58	58	0	1
177582	PRKACA	chr19	14203899	14204074	2	176	176	0	1
177579	PRKACA	chr19	14204415	14204629	2	215	215	0	1
177580	PRKACA	chr19	14208148	14208320	2	173	173	0	1
177585	PRKACA	chr19	14208366	14208511	2	146	146	0	1
177578	PRKACA	chr19	14208551	14208727	2	177	177	0	1
177577	PRKACA	chr19	14211613	14211745	2	133	133	0	1
177587	PRKACA	chr19	14213603	14213751	1	149	149	0	1
177586	PRKACA	chr19	14217547	14217725	2	179	179	0	1
177583	PRKACA	chr19	14218135	14218246	1	112	112	0	1
177581	PRKACA	chr19	14218498	14218821	3	324	324	0	1
177576	PRKACA	chr19	14224921	14224992	2	72	72	0	1
177584	PRKACA	chr19	14228289	14228384	2	96	84	12	0,875
693084	HDAC9	chr7	18201923	18201997	1	75	75	0	1
564675	HDAC9	chr7	18498440	18498549	1	110	110	0	1
564685	HDAC9	chr7	18535860	18535972	1	113	113	0	1
564686	HDAC9	chr7	18624879	18625170	2	292	292	0	1

564678	HDAC9	chr7	18629934	18630134	1	201	201	0	1
564681	HDAC9	chr7	18631114	18631290	1	177	177	0	1
564672	HDAC9	chr7	18633506	18633677	1	172	172	0	1
564688	HDAC9	chr7	18668948	18669129	2	182	182	0	1
564689	HDAC9	chr7	18674225	18674390	2	166	166	0	1
564680	HDAC9	chr7	18684269	18684441	1	173	173	0	1
564665	HDAC9	chr7	18687383	18687646	2	264	264	0	1
564691	HDAC9	chr7	18688064	18688331	2	268	268	0	1
693086	HDAC9	chr7	18705811	18706175	3	365	365	0	1
564663	HDAC9	chr7	18767178	18767405	2	228	228	0	1
564671	HDAC9	chr7	18788603	18788786	2	184	184	0	1
564690	HDAC9	chr7	18801755	18801925	1	171	171	0	1
564662	HDAC9	chr7	18806704	18806803	2	100	100	0	1
564666	HDAC9	chr7	18832943	18833100	2	158	158	0	1
564679	HDAC9	chr7	18868759	18868864	1	106	106	0	1
564677	HDAC9	chr7	18869059	18869196	1	138	138	0	1
564684	HDAC9	chr7	18875065	18875234	1	170	170	0	1
564664	HDAC9	chr7	18875498	18875645	2	148	148	0	1
564667	HDAC9	chr7	18914076	18914244	1	169	169	0	1
564682	HDAC9	chr7	18975407	18975590	1	184	184	0	1
693087	HDAC9	chr7	18993744	18993901	1	158	158	0	1
564693	HDAC9	chr7	19015404	19015601	1	198	198	0	1
693089	HDAC9	chr7	19035621	19035710	1	90	90	0	1
140535	SCNN1B	chr16	23359896	23360256	3	361	361	0	1
140540	SCNN1B	chr16	23364097	23364420	2	324	324	0	1
140534	SCNN1B	chr16	23366595	23366835	2	241	241	0	1
140532	SCNN1B	chr16	23379152	23379305	1	154	154	0	1
140538	SCNN1B	chr16	23382595	23382808	3	214	214	0	1
140539	SCNN1B	chr16	23383072	23383229	2	158	158	0	1
140536	SCNN1B	chr16	23387034	23387201	2	168	168	0	1

140533	SCNN1B	chr16	23388461	23388586	2	126	126	0	1
140531	SCNN1B	chr16	23388625	23388732	2	108	108	0	1
140530	SCNN1B	chr16	23390002	23390113	2	112	112	0	1
140541	SCNN1B	chr16	23391390	23391515	2	126	126	0	1
140537	SCNN1B	chr16	23391717	23392147	3	431	431	0	1
90474	ZNRF3	chr22	29279730	29280079	2	350	350	0	1
90477	ZNRF3	chr22	29383039	29383214	1	176	176	0	1
90473	ZNRF3	chr22	29438458	29438582	1	125	125	0	1
90476	ZNRF3	chr22	29439262	29439443	1	182	182	0	1
90472	ZNRF3	chr22	29440743	29440903	2	161	161	0	1
90471	ZNRF3	chr22	29442679	29442896	2	218	218	0	1
90470	ZNRF3	chr22	29444352	29444504	1	153	153	0	1
90475	ZNRF3	chr22	29445160	29446961	10	1802	1802	0	1
90478	ZNRF3	chr22	29449542	29449635	1	94	94	0	1
177659	ARMC5	chr16	31469693	31469847	2	155	155	0	1
177656	ARMC5	chr16	31470150	31470322	3	173	173	0	1
177660	ARMC5	chr16	31470598	31470686	1	89	89	0	1
177651	ARMC5	chr16	31470764	31471345	5	582	582	0	1
177654	ARMC5	chr16	31473218	31473375	1	158	158	0	1
177653	ARMC5	chr16	31473427	31474263	5	837	837	0	1
177661	ARMC5	chr16	31475690	31476547	6	858	858	0	1
177657	ARMC5	chr16	31477146	31477328	1	183	183	0	1
177655	ARMC5	chr16	31477375	31478235	5	861	861	0	1
31549	CTNNB1	chr3	41265535	41265597	1	63	63	0	1
31556	CTNNB1	chr3	41265992	41266269	2	278	278	0	1
31551	CTNNB1	chr3	41266420	41266723	2	304	304	0	1
31553	CTNNB1	chr3	41266800	41267088	2	289	289	0	1
31552	CTNNB1	chr3	41267126	41267377	2	252	252	0	1
31554	CTNNB1	chr3	41268674	41268868	1	195	195	0	1
31546	CTNNB1	chr3	41274807	41274960	2	154	154	0	1

31548	CTNNB1	chr3	41274995	41275383	3	389	389	0	1
31545	CTNNB1	chr3	41275605	41275813	1	209	209	0	1
31543	CTNNB1	chr3	41277190	41277359	2	170	170	0	1
31555	CTNNB1	chr3	41277815	41278015	1	201	201	0	1
31544	CTNNB1	chr3	41278054	41278225	1	172	172	0	1
31550	CTNNB1	chr3	41279482	41279592	1	111	111	0	1
31547	CTNNB1	chr3	41280600	41280858	2	259	259	0	1
55754	GNAS	chr20	57415137	57415924	6	788	788	0	1
55736	GNAS	chr20	57428296	57430413	14	2118	2118	0	1
55742	GNAS	chr20	57466757	57466945	2	189	189	0	1
55756	GNAS	chr20	57470642	57470764	1	123	123	0	1
55762	GNAS	chr20	57473971	57474065	1	95	95	0	1
55764	GNAS	chr20	57474979	57475035	1	57	57	0	1
55760	GNAS	chr20	57478558	57478665	1	108	108	0	1
55748	GNAS	chr20	57478702	57478871	2	170	170	0	1
55766	GNAS	chr20	57480413	57480560	1	148	148	0	1
55750	GNAS	chr20	57484192	57484296	1	105	105	0	1
55758	GNAS	chr20	57484380	57484503	3	124	124	0	1
55744	GNAS	chr20	57484551	57484659	2	109	109	0	1
55752	GNAS	chr20	57484714	57484884	3	171	171	0	1
55732	GNAS	chr20	57484981	57485161	2	181	181	0	1
55738	GNAS	chr20	57485364	57485481	1	118	118	0	1
55730	GNAS	chr20	57485713	57485909	2	197	197	0	1
173534	PRKAR1A	chr17	66511516	66511742	2	227	227	0	1
82985	PRKAR1A	chr17	66518872	66519092	2	221	221	0	1
82983	PRKAR1A	chr17	66519841	66519982	1	142	142	0	1
82980	PRKAR1A	chr17	66520132	66520243	1	112	112	0	1
82978	PRKAR1A	chr17	66521028	66521124	1	97	97	0	1
82974	PRKAR1A	chr17	66521870	66522078	2	209	209	0	1
82976	PRKAR1A	chr17	66523956	66524066	1	111	111	0	1

82981	PRKAR1A	chr17	66524986	66525157	2	172	172	0	1
82987	PRKAR1A	chr17	66526036	66526167	1	132	132	0	1
173532	PRKAR1A	chr17	66526393	66526615	2	223	223	0	1
173533	PRKAR1A	chr17	66547200	66547290	1	91	91	0	1
177504	PDE8B	chr5	76506726	76507114	3	389	389	0	1
177509	PDE8B	chr5	76607794	76607903	1	110	110	0	1
177513	PDE8B	chr5	76621339	76621579	2	241	241	0	1
177506	PDE8B	chr5	76624798	76624907	1	110	110	0	1
177507	PDE8B	chr5	76627202	76627309	1	108	108	0	1
177523	PDE8B	chr5	76633027	76633165	1	139	139	0	1
177514	PDE8B	chr5	76640653	76640781	1	129	129	0	1
177503	PDE8B	chr5	76645219	76645409	2	191	191	0	1
177511	PDE8B	chr5	76646865	76647003	1	139	139	0	1
177510	PDE8B	chr5	76649146	76649256	1	111	111	0	1
177518	PDE8B	chr5	76696048	76696140	1	93	93	0	1
177512	PDE8B	chr5	76700520	76700647	1	128	128	0	1
177520	PDE8B	chr5	76703181	76703307	1	127	127	0	1
177505	PDE8B	chr5	76704693	76704907	2	215	215	0	1
177517	PDE8B	chr5	76707476	76707571	1	96	96	0	1
177519	PDE8B	chr5	76707900	76708085	1	186	186	0	1
177508	PDE8B	chr5	76708911	76709159	2	249	249	0	1
177516	PDE8B	chr5	76714029	76714296	2	268	268	0	1
177524	PDE8B	chr5	76715567	76715737	1	171	171	0	1
177515	PDE8B	chr5	76717621	76717838	2	218	218	0	1
177522	PDE8B	chr5	76721567	76721746	2	180	180	0	1
177521	PDE8B	chr5	76722245	76722404	2	160	160	0	1
1007754	PRKACB	chr1	84543984	84544079	2	96	96	0	1
1007755	PRKACB	chr1	84610020	84610256	2	237	237	0	1
1007752	PRKACB	chr1	84630071	84630130	1	60	60	0	1
1007758	PRKACB	chr1	84630665	84630721	1	57	57	0	1

746079	PRKACB	chr1	84638987	84639060	2	74	74	0	1
746070	PRKACB	chr1	84640691	84640764	1	74	74	0	1
746081	PRKACB	chr1	84641457	84641515	1	59	59	0	1
746074	PRKACB	chr1	84644835	84644946	2	112	112	0	1
1007756	PRKACB	chr1	84647318	84647436	1	119	119	0	1
746077	PRKACB	chr1	84647858	84648036	1	179	179	0	1
746073	PRKACB	chr1	84649695	84649843	2	149	149	0	1
746080	PRKACB	chr1	84650758	84650890	1	133	133	0	1
746076	PRKACB	chr1	84662274	84662450	2	177	177	0	1
746065	PRKACB	chr1	84663387	84663532	1	146	146	0	1
746069	PRKACB	chr1	84668341	84668513	1	173	173	0	1
1007753	PRKACB	chr1	84670137	84670198	1	62	62	0	1
746067	PRKACB	chr1	84679811	84680025	2	215	215	0	1
1007757	PRKACB	chr1	84700838	84701013	1	176	176	0	1
17904	APC	chr5	112043390	112043604	2	215	215	0	1
17901	APC	chr5	112090563	112090747	2	185	185	0	1
17905	APC	chr5	112101998	112102132	1	135	135	0	1
17902	APC	chr5	112102861	112103112	2	252	252	0	1
17903	APC	chr5	112111301	112111459	1	159	159	0	1
17906	APC	chr5	112116462	112116625	2	164	164	0	1
17899	APC	chr5	112128118	112128251	1	134	134	0	1
17908	APC	chr5	112136951	112137105	1	155	155	0	1
17900	APC	chr5	112151167	112151315	1	149	149	0	1
17913	APC	chr5	112154638	112155066	3	429	429	0	1
17911	APC	chr5	112157568	112157713	1	146	146	0	1
17912	APC	chr5	112162780	112162969	2	190	190	0	1
17910	APC	chr5	112163601	112163728	1	128	128	0	1
17914	APC	chr5	112164528	112164694	2	167	167	0	1
17907	APC	chr5	112170623	112170887	3	265	265	0	1
17909	APC	chr5	112173225	112179848	33	6624	6624	0	1

746913	ATP1A1	chr1	116916109	116916170	1	62	62	0	1
746914	ATP1A1	chr1	116916827	116916888	1	62	62	0	1
690249	ATP1A1	chr1	116926611	116926771	1	161	161	0	1
690246	ATP1A1	chr1	116927380	116927489	1	110	110	0	1
690264	ATP1A1	chr1	116929885	116930138	2	254	254	0	1
690244	ATP1A1	chr1	116930728	116930891	1	164	164	0	1
690251	ATP1A1	chr1	116931235	116931419	1	185	185	0	1
690253	ATP1A1	chr1	116931499	116931666	1	168	168	0	1
690252	ATP1A1	chr1	116932036	116932354	2	319	319	0	1
690250	ATP1A1	chr1	116932810	116933058	2	249	249	0	1
690257	ATP1A1	chr1	116933379	116933538	1	160	160	0	1
690259	ATP1A1	chr1	116935451	116935635	2	185	185	0	1
690256	ATP1A1	chr1	116936128	116936370	2	243	243	0	1
690243	ATP1A1	chr1	116937707	116937932	2	226	226	0	1
690260	ATP1A1	chr1	116939195	116939381	1	187	187	0	1
690261	ATP1A1	chr1	116940485	116940685	1	201	201	0	1
690258	ATP1A1	chr1	116941218	116941436	2	219	219	0	1
690254	ATP1A1	chr1	116941527	116941731	1	205	205	0	1
690248	ATP1A1	chr1	116942017	116942190	1	174	174	0	1
690263	ATP1A1	chr1	116943458	116943653	2	196	196	0	1
690247	ATP1A1	chr1	116943727	116943907	2	181	181	0	1
690265	ATP1A1	chr1	116944151	116944302	1	152	152	0	1
690266	ATP1A1	chr1	116946481	116946622	2	142	142	0	1
746912	ATP1A1	chr1	116947013	116947091	1	79	79	0	1
128169	KCNJ5	chr11	128781144	128782130	6	987	987	0	1
128170	KCNJ5	chr11	128786279	128786651	3	373	373	0	1
177645	NR3C1	chr5	142658904	142659001	1	98	98	0	1
177635	NR3C1	chr5	142661429	142661631	2	203	203	0	1
177648	NR3C1	chr5	142662108	142662315	2	208	208	0	1
177633	NR3C1	chr5	142674992	142675180	2	189	189	0	1

177638	NR3C1	chr5	142678208	142678402	1	195	195	0	1
177646	NR3C1	chr5	142680025	142680353	2	329	329	0	1
177637	NR3C1	chr5	142689637	142689803	2	167	167	0	1
177647	NR3C1	chr5	142693539	142693758	2	220	220	0	1
177639	NR3C1	chr5	142779196	142780429	7	1234	1234	0	1
190752	CYP11B2	chr8	143993371	143993534	2	164	164	0	1
149168	CYP11B2	chr8	143993921	143994168	3	248	248	0	1
149176	CYP11B2	chr8	143994198	143994326	1	129	129	0	1
149173	CYP11B2	chr8	143994676	143994892	2	217	217	0	1
149170	CYP11B2	chr8	143995655	143995859	2	205	205	0	1
149165	CYP11B2	chr8	143996096	143996349	2	254	254	0	1
149172	CYP11B2	chr8	143996437	143996686	2	250	250	0	1
149175	CYP11B2	chr8	143998450	143998655	2	206	206	0	1
190753	CYP11B2	chr8	143998993	143999281	2	289	289	0	1
746924	ATP2B3	chrX	152801681	152801938	3	258	258	0	1
739479	ATP2B3	chrX	152806792	152807039	2	248	248	0	1
739478	ATP2B3	chrX	152807102	152807409	3	308	308	0	1
739476	ATP2B3	chrX	152807756	152807931	2	176	176	0	1
739493	ATP2B3	chrX	152808476	152808651	2	176	176	0	1
739477	ATP2B3	chrX	152811521	152811612	1	92	92	0	1
739480	ATP2B3	chrX	152813268	152813482	2	215	215	0	1
739487	ATP2B3	chrX	152814073	152814337	2	265	265	0	1
739489	ATP2B3	chrX	152814930	152815222	2	293	293	0	1
739490	ATP2B3	chrX	152815478	152815769	2	292	292	0	1
739492	ATP2B3	chrX	152818468	152818752	2	285	285	0	1
739475	ATP2B3	chrX	152821482	152821711	2	230	230	0	1
739486	ATP2B3	chrX	152821764	152821901	2	138	138	0	1
739484	ATP2B3	chrX	152822350	152822506	1	157	157	0	1
739488	ATP2B3	chrX	152823545	152823786	2	242	242	0	1
739481	ATP2B3	chrX	152825162	152825425	3	264	264	0	1

739491	ATP2B3	chrX	152826109	152826370	2	262	262	0	1
739485	ATP2B3	chrX	152827568	152827725	2	158	158	0	1
739495	ATP2B3	chrX	152830354	152830586	2	233	233	0	1
739483	ATP2B3	chrX	152835036	152835239	1	204	204	0	1
746926	ATP2B3	chrX	152845411	152845781	3	371	371	0	1
177536	PDE11A	chr2	178494110	178494315	2	206	206	0	1
177549	PDE11A	chr2	178528569	178528702	1	134	134	0	1
177543	PDE11A	chr2	178534196	178534320	1	125	125	0	1
177558	PDE11A	chr2	178540158	178540271	1	114	114	0	1
177539	PDE11A	chr2	178545529	178545656	1	128	128	0	1
177550	PDE11A	chr2	178562035	178562185	1	151	151	0	1
177546	PDE11A	chr2	178565824	178565964	1	141	141	0	1
177551	PDE11A	chr2	178576472	178576631	1	160	160	0	1
177541	PDE11A	chr2	178592361	178592518	1	158	158	0	1
177555	PDE11A	chr2	178592729	178592925	2	197	197	0	1
177544	PDE11A	chr2	178634026	178634126	1	101	101	0	1
177554	PDE11A	chr2	178681531	178681673	1	143	143	0	1
177547	PDE11A	chr2	178682560	178682677	1	118	118	0	1
177542	PDE11A	chr2	178684922	178685047	2	126	126	0	1
177556	PDE11A	chr2	178704953	178705135	1	183	183	0	1
177538	PDE11A	chr2	178740561	178740675	2	115	115	0	1
177540	PDE11A	chr2	178762760	178762950	2	191	191	0	1
177557	PDE11A	chr2	178769800	178769939	1	140	140	0	1
177552	PDE11A	chr2	178879004	178879212	2	209	209	0	1
177537	PDE11A	chr2	178936228	178937189	6	962	962	0	1
177545	PDE11A	chr2	178969004	178969215	2	212	212	0	1
131475	CLCN2	chr3	184064369	184064613	2	245	245	0	1
68788	CLCN2	chr3	184064698	184064834	1	137	137	0	1
68791	CLCN2	chr3	184069776	184069930	2	155	155	0	1
68795	CLCN2	chr3	184070056	184070144	2	89	89	0	1

68786	CLCN2	chr3	184070206	184070309	1	104	104	0	1
68790	CLCN2	chr3	184070500	184070623	1	124	124	0	1
68784	CLCN2	chr3	184070796	184070960	1	165	165	0	1
68807	CLCN2	chr3	184071013	184071235	2	223	223	0	1
68794	CLCN2	chr3	184071425	184071608	2	184	184	0	1
68798	CLCN2	chr3	184071864	184072127	2	264	264	0	1
68797	CLCN2	chr3	184072311	184072471	2	161	161	0	1
68805	CLCN2	chr3	184072667	184072786	2	120	120	0	1
68787	CLCN2	chr3	184073137	184073342	1	206	206	0	1
68803	CLCN2	chr3	184073457	184073591	1	135	135	0	1
68806	CLCN2	chr3	184074756	184074907	2	152	152	0	1
68802	CLCN2	chr3	184074945	184075079	2	135	135	0	1
68800	CLCN2	chr3	184075125	184075300	2	176	176	0	1
68792	CLCN2	chr3	184075383	184075511	2	129	129	0	1
68799	CLCN2	chr3	184075542	184075669	2	128	128	0	1
68804	CLCN2	chr3	184075725	184075908	2	184	184	0	1
68789	CLCN2	chr3	184075945	184076123	2	179	179	0	1
68796	CLCN2	chr3	184076445	184076626	2	182	182	0	1
68793	CLCN2	chr3	184076738	184076944	2	207	207	0	1
131476	CLCN2	chr3	184079180	184079292	1	113	113	0	1
