# Supplementary material for: Germline targeted next-generation sequencing in patients with adrenal incidentalomas
Source: Front Endocrinol (Lausanne). 2025 Oct 2;16:1685220. doi: 10.3389/fendo.2025.1685220 (PMC12527831; doi:10.3389/fendo.2025.1685220)
Supplement: Supplementary file 3 [file DataSheet3.pdf]

## Sanger Sequencing

Sanger sequencing was performed following standard protocol of the laboratory. In brief, the amplification of DNA (30ng) was carried out through a polymerase chain reaction (PCR) with the AmpliTaq Gold 360 DNA Polymerase (Applied Biosystems, Foster City, CA, USA) employing specific primers and conditions reported in Table *S3*. Subsequently, the PCR products were purified using Exonuclease I and Shrimp Alkaline Phosphatase (ExoSAP) (Applied Biosystems™, Foster City, CA, USA), and sequenced by Big Dye Terminator V3.1 Cycle Sequencing Kit (Applied Biosystems™, Foster City, CA, USA). Then, sequenced products were purified using the Big Dye X Terminator Purification kit (Applied Biosystems™) according to manufacturer's instructions. The sequencing was carried out on the 3500 Dx Genetic Sequencer (Thermo Fisher Scientific, Waltham, Massachusetts, USA) and results were visualized using Sequencing Analysis software v5.4 (Thermo Fisher Scientific, Waltham, Massachusetts, USA).

Table S3. Sanger sequencing PCR conditions and reagents

| Gene | Exon | Coding | Forward | Reverse |
|---|---|---|---|---|
| *APC* | 16 | c.3920T>A | GAAATAGGATGTAATCAGACGA | AGTCTGCTGGATTTGGTTCTA |
| | | c.6422G>C | AGCTATTCAGGAAGGTGCAAA | TGTTTGCTTGAAGGGGCTGT |
| *ARMC5* | 6 | c.2192C>G | CTCCCTTTCCTGCCTCCAAG | ATCTGGGCTTCTGCAAAGCT |
| *CACNA1H* | 2 | c.212C>G | GTGTCACCCTCCGAGAGC | CGGGAAGGATATGGGTTGCA |
| | 10 | c.2246C>T | GCTGAAGAGCTGCCCGTA | CAGCGTGTTGACAAGGATGG |
| | 19 | c.3941A>G | ACGTGGTCCTCGTCTTCATC | GGCCGTGAAGATGTAATTGG |
| | 28 | c.5024G>A | CTGGACGAGGCCCTCAAGTA | CAGACCTCCGTGGGAAAGC |
| *KCNJ5* | 3 | c.977A>G | GCATGTAACTTCCGTTTCCCC | CCCTCTTCATTTCTGCCAGC |
| *PDE11A* | 12 | c.1973A>G | CCTGCACACCTCATGGGATT | GCCTGCCAGTTCCTACGAAT |
| *SCNN1B* | 5 | c.857C>T | TGCAGCTGATGCTGTTTCTT | CTCCTCTTGATGCCTTGGTC |
| *ZNRF3* | 8 | c.1828_1829ins | GACTGCACTGAGGTCAGCAA | CCCAGCACCATGACTGTG |
| | 8 | c.2460del | GGGGTGAAATACGAGGGTCT | TCACAGTCCACATCCTCTGG |

| Reagents and thermal profiles | | | | |
|---|---|---|---|---|
| **Reagents** | for reaction | Temperature | Time | Cycles |
| **AmpliTaq Gold 360 Buffer, 10X** | 2,50 µl | **95 °C** | 00:10:00 | |
| **25 mM Magnesium Chloride** | 2,00 µl | **95 °C** | 00:00:30 | |
| **dNTP mix** | 1,00 µl | **62 °C** *CACNA1H* (c.5024G>A) | | |
| **Primer Forward [0.4µM]** | 1,00 µl | **62 °C** *PDE11A* (c.1973A>G) | | |
| **Primer Reverse [0.4µM]** | 1,00 µl | **60 °C** *APC* (c.3920T>A); (c.6422G>C) | | |
| **BSA (0.2ug/ul)** | 0,50 µl | **60 °C** *ARMC5* (c.2192C>G) | 00:00:30 | 30X |
| **360 GC Enhancer** | 0,50 µl | **60 °C** *CACNA1H* (c. 212C>G) | | |
| **AmpliTaq Gold 360 DNA Polymerase** | 0,50 µl | **60 °C** *KCNJ5* (c.977A>G) | | |
| **Nuclease free water** | 11,00 µl | | | |
| **DNA template (30ng)** | 5,0 µl | **59 °C** *ZNRF3* (c.1828_1829ins); (c.2460del) | | |
| | | **59 °C** *CACNA1H* (c.2246C>T) | | |
| | | **58 °C** *CACNA1H* (c.3941A>G) | | |
| | | **58 °C** *SCNN1B* (c.857C>T) | | |
| | | **72 °C** | 00:00:45 | |
| | | **72 °C** | 00:07:00 | |
| | | **4 °C** | Hold | |
